# Laparoscopic cholecystectomy after endoscopic trans-papillary gallbladder stenting for acute cholecystitis: a pilot study of surgical feasibility

**DOI:** 10.1186/s12893-021-01182-7

**Published:** 2021-04-07

**Authors:** Fumihiro Kawano, Ryuji Yoshioka, Yu Gyoda, Hirofumi Ichida, Tomoya Mizuno, Shigeto Ishii, Toshio Fujisawa, Hiroshi Imamura, Yoshihiro Mise, Hiroyuki Isayama, Akio Saiura

**Affiliations:** 1 Department of Hepatobiliary-Pancreatic Surgery, Juntendo University Graduate School of Medicine, 2-1-1 Hongo, Bunkyo-ku, Tokyo, 113-8421 Japan; 2 Department of Gastroenterology, Juntendo University Graduate School of Medicine, Tokyo, Japan

**Keywords:** Acute cholecystitis, Gallbladder drainage, Endoscopic trans-papillary gallbladder stenting, Laparoscopic cholecystectomy, Percutaneous transhepatic gallbladder drainage, Elective surgery

## Abstract

**Background:**

Percutaneous transhepatic gallbladder drainage (PTGBD) is indicated for patients with acute cholecystitis (AC) who are not indicated for urgent surgery, but external tubes reduce quality of life (QOL) while waiting for elective surgery. The objective of the present study was to investigate the feasibility of laparoscopic cholecystectomy after endoscopic trans-papillary gallbladder stenting (ETGBS) comparing with after PTGBD.

**Methods:**

Intraoperative and postoperative outcomes of patients with ETGBS and PTGBD were retrospectively compared.

**Results:**

Eighteen ETGBS and ten PTGBD patients were compared. Differences in the duration of ETGBS and PTGBD [median 209 min (range 107–357) and median 161 min (range 130–273), respectively, *P* = 0.10], median blood loss [ETGBS 2 (range 2–180 ml) and PTGBD 24 (range 2–100 ml), *P* = 0.89], switch to laparotomy (ETGBS 11% and PTGBD 20%, *P* = 0.52), and median postoperative hospital stay [ETGBS 8 (range 4–24 days) and ETGBS 8 (range 4–16 days), *P* = 0.99]. Thickening of the cystic duct that occurred in 60% of the ETGBS patients and none of the PTGBD patients (*P* = 0.005) interfered with closure of the duct by clipping. No obstruction occurred in ETGBS patients.

**Conclusion:**

ETGBS did not make laparoscopic cholecystectomy less feasible than after PTGBD. This is a pilot study, and further investigations are needed to validate the results of the present study.

## Background

Gallbladder stones are present in nearly 90% of patients who experience acute cholecystitis (AC) [1, 2]. Open surgery or laparoscopic cholecystectomy (LC), which has a shorter hospital stay, are standard treatments. Conservative treatment with antibiotics or gallbladder drainage may be chosen based on the condition of the patient or the severity of the AC. Percutaneous transhepatic gallbladder drainage (PTGBD), an effective and safe drainage procedure, has been used since the 1970s [3–5] but requires external tubes that might decrease the quality of life (QOL) during the waiting period before elective surgery can be performed. As a drainage tool for AC, endoscopic trans-papillary gallbladder stenting (ETGBS) has been reported as an alternative to PTGBD [6–8]. Some reports have focused on the short-term outcomes after LC with gallbladder drainage including both PTGBD and ETGBS, however no reports have addressed the feasibility of LC for AC after ETGBS comparing with LC after PTGBD [9–13]. The objective of the present study was to evaluate the feasibility of LC after ETGBS compared with PTGBD. The present study was considered as pilot study due to immature and small sample size from single institution.

## Methods

### Study population

**Fig. 1:**
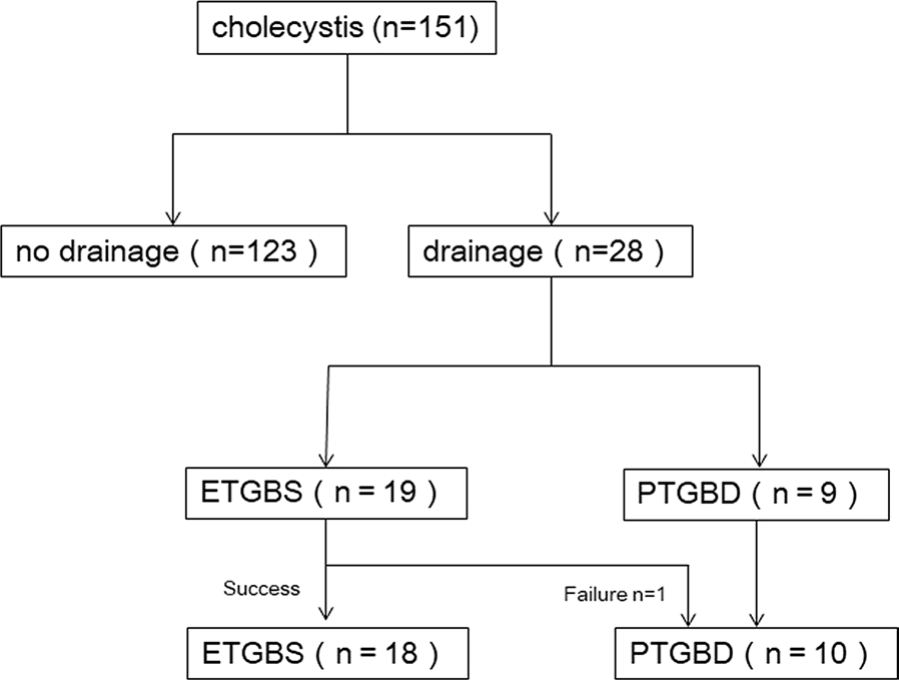
Patient flow diagram

A prospectively maintained database revealed that 240 patients underwent cholecystectomy between January 2017 and March 2019 in our institution. After excluding the patients who underwent open cholecystectomy, LC for chronic cholecystitis, or gallbladder polyp, 151 patients who underwent LC for AC were identified. Of these, 28 patients who underwent LC for AC after gallbladder drainage were included into the analyses (Fig. 1). The study was approved by the Institutional Review Board of Juntendo University Graduate School of Medicine (no. 19-138) and performed following the ethical guidelines for clinical studies.

### Treatment

Definite diagnosis of AC was made by physical examination, laboratory findings, and evidence of inflammation of gall bladder confirmed by sonography and computed tomography. Magnetic resonance imaging was also performed basically. Although we recognized LC as the standard of treatment for AC, urgent or semi-urgent LC for AC had not undergone basically during the study period based on the treatment criteria as previously reported [14]. However, since April 2019, urgent LC has been indicated for the patients with AC who are tolerant for surgery. Prior to January 2017, PTGBD was the sole drainage method for acute cholecystitis in our institution. ETGBS was first adopted in January 2017. It has become the first choice for gallbladder drainage in our institution because we consider that an internalized tube of ETGBS has the advantage of maintaining the patient’s quality of life during the waiting time prior to surgery when compared with the externalized tube of PTGBD. PTGBD was selected when endoscopy was not preferred because of technical difficulties, shortage of skilled labor, or the patient’s condition. One patient in this series was treated by PTGBD at another hospital. Of the 151 study patients, 123 (71%) were managed by conservative treatment. The remaining 28 patients (19%) underwent gallbladder drainage, 18 by ETGBS, and 10 by PTGBD. Elective LC was basically performed 2–4 months after the administration of antibiotics or from performing drainage.

### Gallbladder drainage

**Fig. 2:**
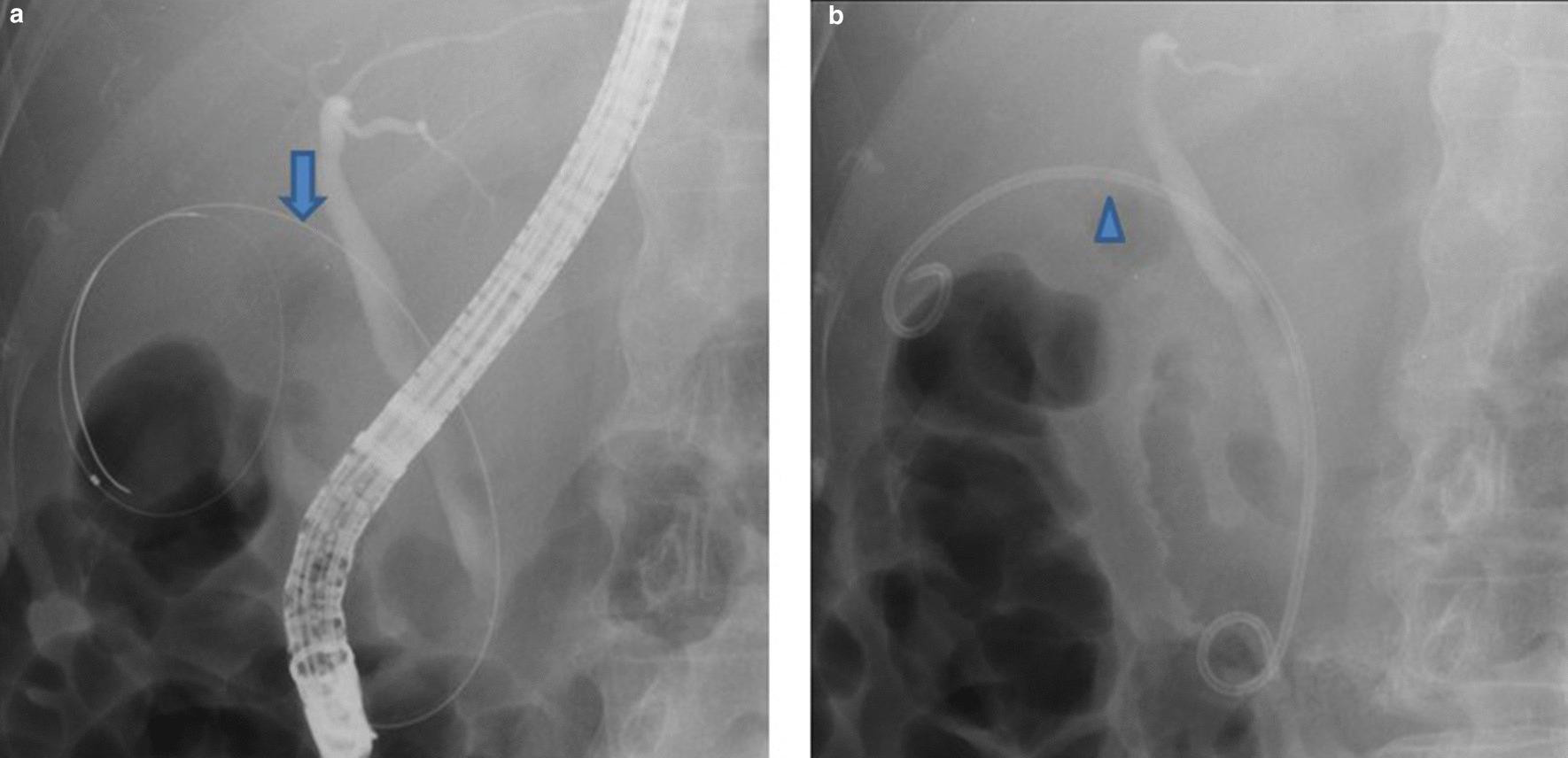
Endoscopic retrograde cholangiopancreatography (ERCP) catheter was inserted into the cystic duct (**a**). The arrowhead shows that the 7 Fr 15-cm double pigtail catheter was inserted into the gallbladder for drainage (**b**)

Except for one PTGBD patient, gallbladder drainage was performed at our institution in the gastroenterology department. PTGBD was performed with ultrasound guidance, drainage was by a pigtail catheter, and cholangiography was performed under fluoroscopy to confirm that the catheter was correctly placed in the gallbladder. ETGBS was performed with sedation following sphincterotomy. Cannulation of the cystic duct was then conducted, followed by trans-papillary placement of a 7 Fr 15-cm double pigtail catheter in the gallbladder (Fig. 2).

### Patient variables

The baseline characteristics and laboratory data findings and intraoperative and postoperative outcomes of the elective LC in ETGBS and PTGBD patients were compared. The severity of AC was determined by the Tokyo guideline criteria [15]. Operation time, estimated blood loss, conversion to open surgery, availability of critical view of safety (CVS), cystic duct closure method, Clavien–Dindo complications, and postoperative hospital stay were the variables that were compared.

### Statistical analysis

Binomial variables were compared using Pearson’s χ^2^ test and Fisher’s exact test. *P*-value of < 0.05 was considered statistically significant. The statistical analysis was performed with JMP 11.2.0 (SAS Institute Inc., Cary, NC, USA).

## Results

Patient status before drainage, severity of AC, and time from drainage to surgery are summarized in Table1. Between-group differences were not significant excluded DIC (*P* = 0.049), but there was a tendency toward more severe AC in the PTGBD group (*P* = 0.08).

**Table 1:** Characteristics of ETGBS and PTGBD patients before gallbladder drainage

Characteristic	ETGBS (n = 18)	PTGBD (n = 10)	*P*
Age	71 (52–88)	77 (48–86)	0.23
Sex (male/female)	15/3	7/3	0.41
Laboratory data before drainage			
WBC (cells/mm^3^)	14650 (4700–36,400)	12,500 (7600–18,300)	0.29
Total bilirubin (mg/dl)	1.7 (0.54–23.44)	0.87 (0.44–3.9)	0.30
Plt (× 10^9^)	18.8 (12–30.9)	29.3 (6.4–31.2)	0.77
Cre (mg/dl)	0.70 (0.33–1.33)	0.87 (0.61–10.33)	0.12
AST (U/l)	41 (17–578)	28 (18–208)	0.48
ALT (U/)	42 (12–490)	35 (8–121)	0.23
ALP (U/l)	215 (159–2368)	239 (133–675)	0.27
Alb (g/dl)	3.2 (2.7–5.1)	3.3 (2.9–4.6)	0.51
CRP(mg/dl)	15.7 (0.05–30.4)	16.9 (0.3–43.7)	0.30
PT–INR	1.11 (0.99–1.43)	1.02 (0.89–1.78)	0.72
DIC			
Yes	0 (0%)	2 (20%)	0.119
No	18 (100%)	8 (80%)	
Ascites			
Yes	0 (0%)	0 (0%)	N.A
No	18 (100%)	10 (100%)	
Anticoagulant therapy			
Yes	4 (22%)	3 (30%)	0.674
No	14 (78%)	7 (70%)	
Time to operation (day)	72 (11–118)	78 (32–202)	0.08
ASA*			
1	5 (28%)	3 (30%)	0.97
2	8 (44%)	4 (40%)	
3	5 (28%)	3 (30%)	
Grade**			
I	12 (67%)	7 (70%)	0.08
II	6 (33%)	1 (10%)	
III	0 (0%)	2 (20%)	

*ASA American Society of Anesthesiologists Physical Status Classification. **Classified by the Tokyo guidelines (17). Data are median (range) or number (%)

The surgical outcomes of the drainage and non-drainage groups are shown in Table 2. There are significant differences between the drainage and non-drainage groups in the variables as follows; operation time, blood loss, conversion to open surgery, closure of cystic duct, and hospital stay, respectively.

**Table 2:** Surgical results in No drainage and drainage patients

	No drainage (n = 123)	Drainage (n = 28)	*P*
Operation time (min)	127(64–417)	209 (107–357)	< 0.0001
Blood loss (ml)	8 (1–195)	21 (2–180)	0.0061
Conversion to open surgery	5(4%)	4(14%)	0.0392
Confirmation of CVS			
Yes	117	20	< 0.0001
No	6	8	
Closure of the cystic duct			
Clipping	117	16	< 0.0001
Ligation	6	9	
ASA			
1	54	8	0.118
2	48	12	
3	17	8	
Postoperative complication Clavien–Dindo criteria			
Grade I	116	24	0.403
Grade II	4	4	
Grade IIIa,b	3	0	
Postoperative stay	5(3–25)	8(4–24)	0.0063

*CVS* critical view of safety

Data are median (range) or number (%)

Acute pancreatitis, perforation, bleeding, or other complications associated with either the ETGBS or the PTGBD procedures were not observed. Out of the 19, 18 ETGBS procedures were successful (95%). One ETGBS patient was switched to PTGBD because cannulation of the cystic duct was not possible (Fig. 1).

Cholecystitis recurred in three patients in the PTGBD group (30%) while waiting for surgery, but none of the patients in the ETGBS group experienced recurrent cholecystitis because of cystic duct occlusion. Intra- and postoperative factors in the ETGBS and the PTGBD group are summarized in Table 3. The median operation times were 209 min (range 107–357) for ETGBS and 161 min (range 130–273) for PTGBD (*P* = 0.10). Switching to open surgery was needed in 11% in of ETGBS procedures and 20% of PTGBD procedures (*P* = 0.52). In each case, the reason for switching to open surgery conversion was severe inflammation. The attainment of CVS was impossible in 5 of the 18 ETGBS patients (28%) and in 3 of 10 PTGBD patients (30%) (*P* = 0.90). Those patients were managed by a bail-out technique that involved fundus-first LC in four patients and conversion to laparotomy in four.

**Table 3:** Surgical results in ETGBS and PTGBD patients

	ETGBS (n = 18)	PTGBD (n = 10)	*P*
Operation time (min)	209 (107–357)	161 (130–273)	0.10
Blood loss (ml)	21 (2–180)	24 (2–100)	0.89
Confirmation of CVS			
Yes	13	7	0.9
No	5	3	
Conversion to open surgery	2 (11%)	2 (20%)	0.52
Closure of the cystic duct			
Clipping	7	8	0.005
Ligation	9	0	
Postoperative complication Clavien–Dindo criteria			
Grade I	15	9	0.63
Grade II	3	1	
Grade III, IV, V	0	0	
Postoperative hospital stay	8 (4–24)	8 (4–16)	0.99

*CVS* critical view of safety

Data are median (range) or number (%)

**Fig. 3:**
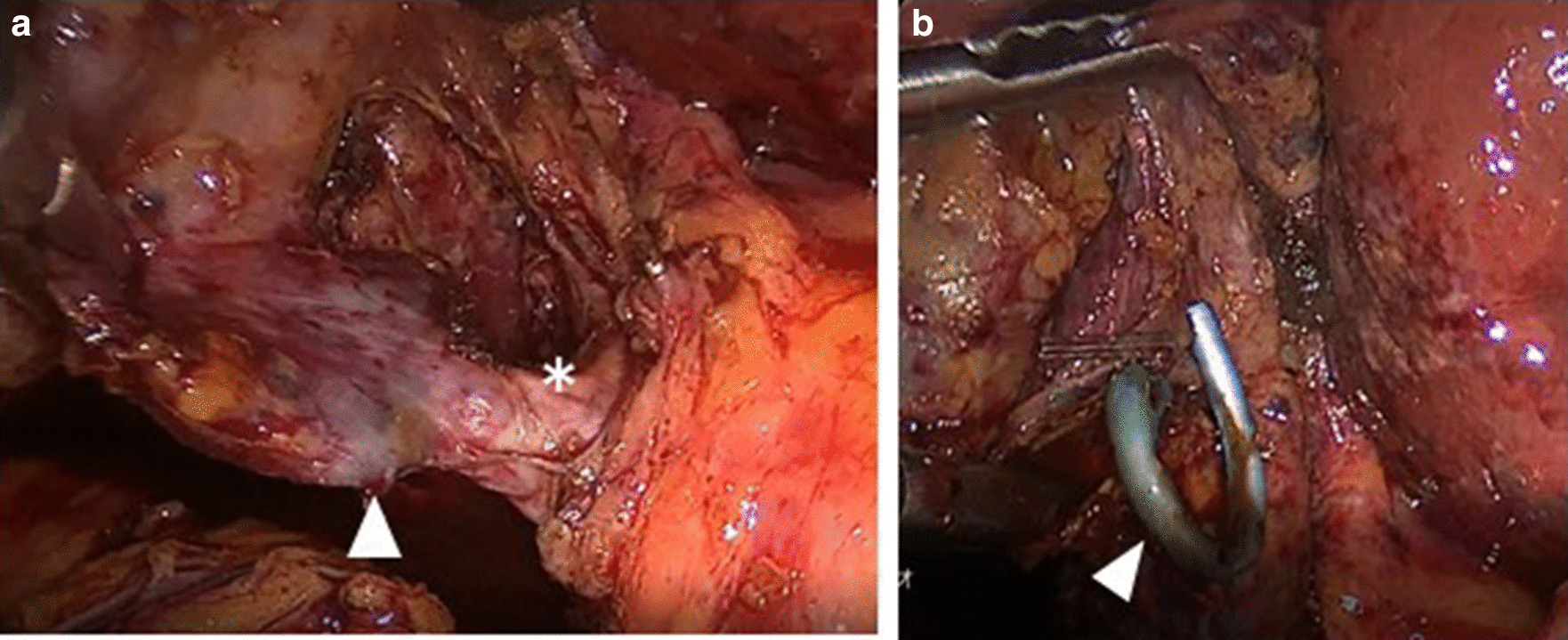
Representative intraoperative images of patients with preoperative ETGBS. **a** Critical view of safety and cystic duct thickening (*). The arrowhead shows the ETGBS from the outside of the duct. **b** The ETGBS tube (arrowhead) was removed via the incision of the cystic duct

The ETGBS tube was easily removed from the cystic duct by making an incision that was ligated with a double 5-mm M-L clip, but in 9 of the 18 cases (50%), thickening of the cystic duct prevented the use of a clip and the duct was closed by ligation using with 2–0 silk or an Endoloop^®^ (PDS^®^II) (ETHICON; NJ, USA). (Fig. 3) The cystic duct was closed with clips in all PTGBD patients *(P* = 0.005). There was no postoperative mortality and Clavien–Dindo Grade III or greater morbidities. There were no significant differences in the postoperative outcomes seen in the two study groups.

## Discussion

After evaluating short-term outcomes, LC was found to be safe in patients with preoperative ETGBS. This is the first report of the evaluation of short-term outcomes focused on after each drainage method for AC of PTGBD vs. ETGBS. The 2018 Tokyo guidelines recommend up-front cholecystectomy for mild or moderate AC [15]. However, we occasionally encounter the situations in which urgent surgery for patients with severe AC could not be performed due to the patients’ comorbidity or lack of medical resources. Gallbladder drainage is often necessary for such patients. PTGBD has been a standard treatment, but we speculate that the external tube causes discomfort during the wait for surgery, and avoiding skin complications requires daily management. The present study could not address this issue due to the lack of objective data about QOL assessment. Objective QOL assessment during the waiting time should be assessed in the future study. Other drainage methods are available. Toyota et al. reported the usefulness of ENGBD for salvage of patients with AC [16]. Waiting time between salvage and LC was reported as 1–3 days. External tube from nasal cavity would impair the patient’s QOL compared with ETGBS. Endoscopic naso-gallbladder drainage (ENGBD) could be another alternative option for gall bladder drainage if waiting time to LC was aimed to be short. Although there have been reports on the safety of elective surgery after drainage [17], there have been no papers that focus on and compare the surgical outcomes of elective surgery after PTCD and ETGBS. This study evaluated the feasibility of laparoscopic cholecystectomy after ETGBS vs. PTGBD.

ETGBS is less likely than PTGBD to cause the recurrent cholecystitis during the waiting time, which is a clinically significant advantage. In fact, no patients with ETGBS experienced recurrent cholecystitis while waiting for surgery, but recurrent cholecystitis did occur in three of the ten with PTGBD (30%). On the other hand, sphincterotomy for patients with ETGBS might impact the increased risk of cholangitis after LC. It should be taken into consideration in the long-term follow up. ETGBS can be performed by avoiding sphincterotomy even when patients have some risk of hemorrhage because of anticoagulant medications or those with disseminated intravascular coagulation (DIC). Bleeding complications are estimated to occur in 1.5–2.7% of PTGBD cases, with an increased bleeding risk in patients with a blood coagulation disorder [16, 18].

There are concerns of operative difficulty in patients with ETGBS because of inflammation around the cystic duct and cannulation of the drainage tube interfering with dissection in Calot’s triangle. In this patient series, ETGBS did cause thickening of the cystic duct and inflammation of Calot’s triangle (Fig. 3) that resulted in half of the patients requiring duct suturing because clip closure was not possible. Thickening of the cystic duct should be taken into consideration during surgery in patients with preoperative ETGBS. However, in this series, ETGBS did not increase the operative difficulty compared with PTGBD. The procedure duration, blood loss, and rates of conversion to open surgery were equivalent.

ETGBS cannot be performed patients in poor condition. Endoscopy of the biliary tract needs sedation that would not be tolerated by patients at risk of shock. In this study, two PTGBD patients had severe Tokyo grade III AC. There were no grade III patients in the ETGBS group. ETGBS cannot be performed patients in poor condition. Endoscopy of the biliary tract needs sedation that would not be tolerated by patients at risk of shock. In this study, two PTGBD patients had severe Tokyo grade III AC. There were no grade III patients in the ETGBS group. Relatively limited indication of ETGBS would be a disadvantage compared with PTGBD. ETGBS is a complex procedure with a reported success rate of 77.3–89.5% [12, 13, 16, 18–20]. In this study, the success rate of ETGBS was 95% (18 of 19 procedures), which is higher than in previous reports. One patient was switched to PTGBD from ETGBS because of incomplete cannulation of the cystic duct despite of the absence of stone incarceration. Procedure time of insertion of ETGBS is considered as longer than that of PTGBD. Although procedure time of ETGBS was not available due to the retrospective nature of the present study, it was reported to be around 35 min [21]. Therefore, we consider that ETGBS is less burdensome than acute surgery for high risk patients. ETGBS-associated complications, including pancreatitis, liver dysfunction, biliary tract injury, and intestinal tract injury, have been reported in about 1.8% of procedures. That rate is similar to that reported for ERCP [16, 22]. No complications associated with ETGBS, including pancreatitis or perforation, occurred in this study. The limitations of this study include the small sample size, inclusion from a single institution, and its retrospective nature. However, to our knowledge, no studies have reported the outcomes of LC in patients with ETGBS compared those with PTGBD. Although this study has some novelty, it should be validated by prospective studies to address the actual feasibility of LC after ETGBS.

## Conclusion

Most patients with AC require emergency drainage, and those taking anticoagulants, with severe inflammation accompanied by DIC or with ascites, do not qualify for PTGBD. ETGBS with trans-papillary cannulation may be indicated for such patients. This pilot study showed that LC was performed successfully and safely after either ETGBS or PTGBD. ETGBS did not make laparoscopic cholecystectomy less feasible than after PTGBD. These findings should be validated by further study with multi-institutional, large sample size.

## Data Availability

The data and materials used and/or analyzed during the current study are available from the corresponding author on reasonable request.

## Abbreviations

PTGBD: Percutaneous transhepatic gallbladder drainage
AC: Acute cholecystitis
ETGBS: Endoscopic trans-papillary gallbladder stenting
QOL: Quality of life
LC: Laparoscopic cholecystectomy
DIC: Disseminated intravascular coagulation
CVS: Critical view of safety
ERCP: Endoscopic retrograde cholangiopancreatography
